# Review on the transmission porcine reproductive and respiratory syndrome virus between pigs and farms and impact on vaccination

**DOI:** 10.1186/s13567-016-0391-4

**Published:** 2016-10-28

**Authors:** Emanuela Pileri, Enric Mateu

**Affiliations:** 1 Departament de Sanitat i Anatomia Animals, Universitat Autònoma de Barcelona, Campus UAB, 08193 Cerdanyola del Vallès, Spain; 2 Centre de Recerca en Sanitat Animal (CReSA)-IRTA. Edifici CReSA, Campus UAB, 08193 Cerdanyola del Vallès, Spain

## Abstract

Porcine reproductive and respiratory syndrome (PRRS) is considered to be one of the most costly diseases affecting intensive pig production worldwide. Control of PRRS is a complex issue and involves a combination of measures including monitoring, diagnosis, biosecurity, herd management, and immunization. In spite of the numerous studies dealing with PRRS virus epidemiology, transmission of the infection is still not fully understood. The present article reviews the current knowledge on PRRSV transmission between and within farm, and the impact of vaccination on virus transmission.

## Introduction

Porcine reproductive and respiratory syndrome (PPRS) has become one of the most important diseases of intensive pig production worldwide. The economic impact of PRRS in breeding and farrowing units is caused mostly by a reduction in the number of weaned pigs and by an impairment of the farrowing rates. Infection in growing-finishing pigs may increase secondary infections and mortality rates, as well as resulting in retarded growth, a high dispersion of weights at slaughter age, and increased antimicrobial usage. In 2005, the total annual cost of PRRS outbreaks in USA was estimated to be about USD 560 million, which comprised USD 67 million for the breeding-farrowing phase, USD 201 million for the nursery phase and USD 292 million for the grower-finisher phase of production [1]. More recently, Holtkamp et al. [2] calculated a cost of USD 663 million/year for the United States, representing a 10% increase compared to Neumann et al. [1]. In Europe, average losses related to PRRS outbreaks were estimated by Nieuwenhuis et al. [3] in 126 €/sow, slightly more than the USD 121/sow reported by Neumann et al. [1].

The negative impact of PRRS on the economic margin of pig production has stimulated the efforts to control and eventually eradicate the disease. It is assumed that control of PRRS virus (PRRSV) relies on four different aspects: early diagnosis and monitoring, biosecurity, herd management, and immunisation. A deep understanding of how the virus is transmitted between animals and how it spreads in pig populations is crucial for choosing the most suitable strategies to cease viral circulation and to avoid reintroduction of the virus into the farm. Therefore, the main objective of the present paper is to review the current knowledge about PRRSV transmission, taking into account the pathogenesis of the infection as well as the host and viral factors that can influence the dynamics of transmission. Additionally, the effect of vaccination on PRRSV transmission and the usefulness of this measure to stop viral circulation are also discussed.

## Virus stability in the environment and disinfection

PRRSV is a small, enveloped, positive-sense, single-stranded RNA virus that is quickly inactivated by lipid solvents, heat, drying, or at a pH below 5 or above 7 [4]. Bloemraad et al. [5] demonstrated that Lelystad virus (LV) is inactivated after 6 min at 56 °C or 3 h at 37 °C, but it is stable for 140 h at 4 °C and for several months in cell culture medium at pH 7.5 at temperatures between −70 and −20 °C. With a genotype 2 isolate, Van Alstine et al. [6] showed similar results, and at 72 h after necropsy of experimentally infected pigs the infectious virus could only be recovered from 7% of the examined tissue samples. Finally, it has been shown in the Midwest of USA that PRRS outbreaks follow a seasonal pattern, with an onset of the epidemic in October [7]. The cause of this seasonal pattern is unclear although it could be in part related to the stability of the virus in the cold season.

As for disinfection, complete inactivation of the virus is accomplished in 1 min using iodine (0.0075%) or quaternary ammonium compounds (0.0063%) [8]. Complete inactivation of PRRSV is also achieved with chlorine, although a higher disinfectant concentration (0.03%) and a longer exposure time (10 min) were needed [8]. Similarly, 10 min of ultraviolet light exposure completely inactivated the virus on common farm surfaces and materials [9].

## Transmission of PRRSV

### Routes and methods of transmission of PRRSV

Pigs can be infected by either direct contact or indirectly through fomites. Exposure to PRRSV occurs by the respiratory and oral routes and through the mucosae or percutaneously. The methods involved are aerial transmission (either short or long distance), by coitus or insemination, ingestion, by contact, and by inoculation (most often iatrogenically). Vertical transmission is important during the last trimester of gestation.

The minimum infectious dose (MID) of PRRSV varies depending on the route of exposure. Hermann et al. [10] evaluated the infectious dose 50 (ID_50_) by oral and nasal exposure. Exposure of pigs to isolate PRRSV 2 isolate VR-2332 resulted in an ID_50_ of 10^5.3^ and 10^4.0^ TCID_50_ for the oral and intranasal route, respectively. The same authors found that inoculating pigs by the intramuscular route, the ID_50_ was 10^2.2^ TCID_50_. In contrast, Yoon et al. [11] reported that ≤10 PRRSV particles of the genotype 2 isolate ISU-P were enough to infect pigs parenterally. Differences in the infectivity among PRRSV isolates were also observed for other transmission routes. Thus, Cutler et al. [12] calculated that the ID_50_ for the aerosol exposure to genotype 2 isolate MN-184 was less than 2 TCID_50_ while Hermann et al. [13] reported an ID_50_ of 10^3.1^ TCID_50_ for the aerosol exposure using isolate VR-2332. Regarding the sexual transmission, Benfield et al. [14] estimated the ID_50_ for exposure via artificial insemination to be 10^3.3^ TCID_50_.

According to the available data, the percutaneous exposure is the route with the lowest MID. On the farm, parenteral exposure could be frequent and would include standard practices such as ear notching, tail docking, teeth clipping, and injection of drugs and vaccines. In the peak of the viraemia, infected animals have a viral load of at least 10^3^ to 10^4^ TCID_50_/mL [15]. Assuming a MID between 10^1^ and 10^2^ TCID_50_ for the percutaneous route, blood volumes of 1–10 µL could be sufficient to produce transmission. In fact, Otake et al. [16] demonstrated that transmission of PRRSV is achieved by using contaminated needles. Likewise, Baker et al. [17] showed that transmission of MN-184 isolate can occur by using the same needle between pigs, and that the use of needle-free injection device (NFID) reduced, but did not fully prevent, this type of transmission. However, the authors were not able to identify the route of transmission in the NFID group although airborne virus was discarded since controls remained negative for the duration of the trial.

Moreover, normal pig behaviour may also result in parental exposure through bites, cuts, scrapes and/or abrasions that occur during fighting between pigs. Bierk et al. [18] demonstrated that aggressive behaviour between infected sows and susceptible contacts may play a role in PRRSV transmission.

### Development of viraemia and persistence in lymphoid tissues

Following exposure to the virus, replication occurs initially in permissive macrophages of lymphoid tissues at the portal of entry and then the virus rapidly spreads throughout the body by the lympho-haematic route. In a genotype 2 model viraemia started as early as 12 h post-infection [19] and viral load peaked in serum around 7–10 days post-infection (dpi). The duration of viraemia varies depending on the PRRSV strain and on the age of the animal [20–23]. This general picture is similar in genotype 1 [22]. In both cases, several studies indicated that in general the period of viraemia may range from a few weeks (usually <4) in adults or grower-finishers to up to three months in very young piglets [23–26]. In adult sows, viremia may be limited to just one week as shown by Karniychuk with genotype 1 PRRSV [27]. When infecting 2-week-old piglets with a genotype 2 isolate, Wills et al. [26] detected viral RNA in serum in 1/28 pigs at 251 dpi, although they could not isolate infectious virus from serum after 56 dpi.

The lungs and the lymphoid organs such as tonsils, Peyer’s patches, thymus, and spleen [15, 28–30] are the tissues with the highest viral loads in the initial phase. In lungs the virus is usually detected from 1 day post-exposure until 28 dpi [30, 31], although persistence of virus in the lungs been described until 49 days post-exposure in young pigs [32].

The viraemic phase of the infection is followed by a period of confinement of the virus in secondary lymphoid tissues, and lower viral replication. Studies carried out with genotype 2 PRRSV showed that the virus could be isolated from oropharyngeal scrapings until 157 dpi [33], and Horter et al. [25] found that 86% of the infected pigs (51/59) carry the virus in oropharyngeal scrapings or tonsils between 63 and 105 dpi. Viral genome can be present in serum and tonsils until 132 days after birth in piglets surviving congenital infection [34, 35]. However, the mere presence of the virus in tissues is not a direct equivalent to transmission. With regards to the potential for transmission, Allende et al. [24] in a bioassay with materials of type 2 infected pigs, showed that at 150 dpi, tonsil tissue of 2/5 contained sufficient infectious virus to be transmitted. Bierk et al. [18] demonstrated that non-viraemic sows were able to transmit the infection by direct contact with PRRSV-naïve sows at 49, 56, and 84 dpi. Likewise, non-viraemic pigs transmitted the virus to naïve sentinels up to 62 dpi [36]. Conversely, non-viraemic sows were still able to transmit the infection by contact with naïve sows at periods between 49 and 86 dpi [18]. Charpin et al. [37] showed that with genotype 1 PRRSV, piglets reach a peak of infectiousness around 9 dpi, then decreasing until 42 dpi. Transmission of PRRSV from congenitally infected piglets to sentinel animals was observed up to 112 days after birth [35]. In summary, it can be assumed that contagiousness decreases with time, but transmission is possible under natural conditions up to 3 months in horizontally infected pigs, while for congenitally infected animals the contagiousness period may well exceed this period.

Regarding the ability of chronically infected pigs to transmit the virus to susceptible animals, it is worth noting that circumstances causing stress such as farrowing, regrouping etc., might induce a reactivation of viral replication and shedding. For example, Albina et al. [38] demonstrated reactivation of PRRSV shedding after corticosteroid treatment at 15 weeks after the initial seroconversion of the animal.

### Viral shedding

The development of viraemia and the body distribution of susceptible macrophages lead to the shedding of PRRSV by multiple routes. In fact, the presence of the virus in nasal secretions, saliva, urine, faeces, mammary gland secretions, and semen is well documented in several studies [19, 39–52].

Regarding nasal shedding, it seems to be strain dependent, at least with genotype 1. For the prototypical type 1 isolate Lelystad virus, Duan et al. [15] showed that nasal shedding was scarce achieving isolation only in 4/8 pigs at 3 days post-inoculation and in 1/8 at 7 days post-inoculation and always at low titres. Charpin et al. [37] using a genotype 1 PRRSV strain indicated that the viral load in nasal secretions of inoculated piglets increased very rapidly, reaching a maximum at 2 dpi, and then decreased steadily until 48 dpi. No RT-PCR positive nasal swabs were detected after 49 dpi [37]. Further work done at the University of Ghent has shown that the ability of a given isolate for infecting different subsets of potentially susceptible cells in the nasal mucosa is the critical factor for nasal shedding [53, 54] (see also Sect. 4.2).

With genotype 2 Rossow et al. [47] reported nasal secretion in only 1.9% (2/105) of the nasal swabs collected from experimentally inoculated pigs during a 28-day-observation period [47], and no virus was isolated in nasal secretions of experimentally infected gnotobiotic pigs [19]. Christianson et al. [39] inoculated sows with genotype 2 virus around 50 days of gestation with the genotype 2 VR-2332 isolate. Shedding by the nasal route was observed from 3 to 9 dpi, while faecal swabs were positive for viral isolation at 2, 4–6, 8, and 9 dpi. Conversely, Yoon et al. [51] described intermittent nasal and faecal shedding until 38 dpi in experimentally inoculated piglets. Rossow et al. [47] isolated VR-2332 only sporadically from faecal swabs of piglets at 28 dpi, whereas the virus was not detected in faeces of experimentally infected gnotobiotic pigs [19]. Shedding in faeces is irregular [47].

Shedding in oral fluids seems to be more constant but most data are restricted to genotype 2 virus. Thus, Wills et al. [50] isolated the virus at least once in 5/6 inoculated pigs (83.3%), and shedding was detected up to 42 dpi, although intermittently [50]. Prickett et al. [46] assessed viral shedding in oral swabs, as well as in pen-based oral fluids, over a 63-day period in pigs inoculated with a genotype 2 PRRSV strain. Oral fluids were positive by RT-PCR from 3 dpi to 4–5 weeks post-inoculation, with sporadic positive results thereafter. Moreover, viral load in serum and oral fluid samples followed a similar pattern, although oral fluids usually had a lower concentration of virus [46]. Conversely, Kittawornrat et al. [44] found that serum contains equal or higher concentration of virus than oral fluids for the first 14 dpi, while the amount of virus was higher in oral fluids from 21 dpi onwards. In all cases, shedding in oral fluids is detected early in the course of infection (76–100% of qRT-PCR positive samples at 2–4 dpi, respectively), regardless of the viral isolate used as inoculum [44].

The presence of virus in oral fluids, and the relative constancy of this shedding over time, may have also important implications in the transmission of PRRSV. As the MID required for the parenteral route is the lowest, common pig behaviours such as fighting, tail-biting, and ear-biting could result in effective transmission. It would be worth studying whether the actions for enhancing pig welfare may result in decreased transmission of PRRSV.

As regards shedding in semen of infected boars, viral genome was detected by RT-PCR as early as 3 dpi, and up to 92 dpi in 1/4 boars inoculated with the VR-2332 isolate [40]. Infectious virus in semen was intermittently detected by viral isolation and/or swine bioassay from 3 up to 43 dpi in experimentally infected boars, although viraemia lasted less than 14 days [40, 41, 45, 48]. Moreover, PRRSV was isolated from the bulbourethral gland of one boar at 101 dpi, suggesting that the male reproductive tract could be a long-term source of the virus, and that viremia is not an adequate indicator of the potential contagiousness of a boar [40].

PPRSV can be also shed in urine [47, 50] and mammary gland secretions [43, 49]. In experimentally infected sows, genotype 2 PRRSV was detected by RT-PCR in the first day of lactation [43]. Wagstrom et al. [49] showed that naïve sows inoculated late in gestation shed PRRSV in colostrum and milk, but only for a limited number of days and in low concentrations, as determined by virus isolation and titration. In addition, vaccination of sows seemed to prevent shedding during subsequent lactations, and the virus was not detected in any of the milk samples collected from 181 sows of 8 endemically infected herds. These results suggest that colostrum and milk can be a source of virus for the offspring, but their contribution to PRRSV transmission is probably secondary [49]. Fecal shedding seems to be irregular.

Infected pigs produce contaminated aerosols when breathing, sneezing or coughing. Cho et al. [55] inoculated two groups of pigs intranasally with the genotype 2 isolates MN-30100 and the MN-184 isolates, respectively. Aerosol samples were then collected on alternate days from 1 to 21 dpi and were analysed by qRT-PCR. Results showed that a small number of pigs inoculated with PRRSV MN-30100 shed intermittently throughout the sampling period, whereas more consistent shedding was observed in a larger number of pigs inoculated with PRRSV MN-184. Although the difference in the mean concentrations of PRRSV RNA in aerosols from pigs infected with PRRSV MN-30100 or PRRSV MN-184 was not significant, the logistic regression analysis showed that inoculation with PRRSV MN-184 resulted in a significantly higher likelihood of aerosol shedding than inoculation with PRRSV MN-30100. These results supported the notion that PRRSV transmission by aerosol is dependent on the PRRSV strain involved.

## Factors influencing PRRSV transmission

In the following sections, the most relevant aspects concerning the factors influencing the PRRSV transmission between pigs are summarised.

### Age of the pigs at the time of the infection

Klinge et al. [21] showed that 3-week-old piglets had significantly longer viraemias than finishers or adult pigs, regardless of the PRRSV isolate used as inoculum. Likewise, 2-month-old pigs had significantly higher viral loads in lymph nodes, lungs, and tracheobronchial swabs than 6-month-old animals, regardless of the virulence of the challenge strain [21, 55]. In addition, Thanawongnuwech et al. [56] showed that pulmonary macrophages from 4-week-old pigs yielded higher virus titres than pulmonary macrophages from 4-month-old pigs. All the previous data present indirect evidence of a higher contagiousness of piglets compared to finishers or adult pigs, although a precise evaluation is lacking. This can be relevant for the control of the infection in farrow-to-finish farms, in particular regarding the potential re-infection of sows or new-born pigs from nurseries.

### Variation in the virulence of PRRSV isolates

Cho et al. [20] showed that inoculation with the genotype 2 highly virulent strain MN-184 resulted in significantly higher viral loads in serum and tonsils than inoculation with the low virulence isolate MN-30100. Also, MN-184-inoculated pigs produced contaminated aerosols for a longer period [55]. Likewise, boars infected with MN-184 isolate showed higher viral titres in serum and shed significantly higher amounts of virus in oral fluids at 7–14 dpi than pigs inoculated with isolates of lesser virulence [44]. Increased replication efficiency and higher viral titres in serum were also observed for the highly pathogenic HuN4 [57] and Lena [52] strains, although in these cases the viral shedding was not assessed. Moreover, in several studies by Frydas et al. [53, 54, 58] it was shown, at least for genotype 1, that different isolates replicate to a different extent in the nasal mucosa, and this may have an influence on the formation of aerosols. Collectively, these data suggest that pigs infected with highly virulent strains could shed higher amounts of virus than pigs infected with low virulent strains, and that the replication in nasal mucosa varies between isolates. However, experiments aimed at comparing the transmissibility of PRRSV strains of different virulence are still lacking.

### Immune response against PRRSV

The immune response against the virus is probably the most important factor influencing the course of the infection, and consequently the susceptibility and infectiousness of pigs. The development of neutralising antibodies (NA) and/or of a cell-mediated immunity (CMI) have been related with the clearance of PRRSV infection or pre-exposure protection [23, 59–63]. Nevertheless, since viraemia can be resolved without the development of neutralising antibodies or strong CMI, the correlates of protection are still not well understood [64–68]. In addition, the genetic/antigenic diversity of PRRSV and the idiosyncrasy of each individual are also known to play a role. In fact, in general terms, both virological and clinical protection is good in the case of homologous challenge [69], whereas it is only partial for heterologous challenges, although genetic similarity between the immunising and the challenge strain is not fully predictive of the degree of protection [59, 70–73]. Although this topic is beyond the scope of the present review, it will be discussed with more details in the Sect. 4.2.

## Transmission and dynamics of the infection within herds

When PRRSV enters into an immunologically naïve herd, all pigs are affected, and a clinical outbreak usually occurs. In a typical farrow-to-finish farm, viral circulation begins in one or more stages of production, commonly in the breeding herd, and then the virus spreads to all production stages in about 2–3 weeks. Sows can transmit the virus to their offspring by the trans-placental route and/or by direct contact during lactation.

Piglets infected congenitally or very early in life can harbour the virus for several months, and can contribute to the spread of the infection in the following productive stages. As the infection progresses, the proportion of immune pigs increases, and that of susceptible animals decreases. This leads to the decline phase of the epidemic in 1–5 months, depending on the herd size and the time needed to achieve a protective immunity in the majority of pigs [74–76]. At this point, the infection usually becomes endemic, although in some small farms it may fade out just because of the exhaustion of susceptible pigs [77]. In fact, the transmissibility of the virus, the duration of the infectious period, and the existence of susceptible pigs in the population mainly determine the spontaneous extinction of a virus in a population. Nodelijk et al. [78] estimated, by using a Monte Carlo simulation, that the average time for type 1 PRRSV to fade-out was about 6 years in a closed herd of 115 sows, whereas it was as long as 80 years in a closed herd of 230 sows. These estimations are in accordance with those of Evans et al. [79] indicating that the persistence of the infection is more likely as the herd size increases, and when the gilt pool is not properly isolated from sows. In addition, PRRSV fade-out seems less likely to occur when the infection is established in the farrowing house and in piglets due to the retrograde transmission from infected nurseries or finishers to the breeding herd [79].

The maintenance of the infection within a farm is basically due to the combination of animals with long-term infection and the continual availability of susceptible pigs. The latter can be added to the population by replacement, by birth of piglets from seronegative sows, by loss of passive immunity in young pigs, or by loss of active immunity in previously infected pigs [77]. As a result, PRRSV can circulate in the farm for several years. For instance, a longitudinal study conducted in a Dutch breeding herd by Nodelijk et al. [78] shown that seroprevalence in sows during an acute outbreak was 86–95%, and that sows that initially escaped the infection did seroconvert at a later stage, indicating the existence of sub-populations that have a low level of viral circulation. In sows, the infection cycle can be maintained by transmission between them, but also by anterograde transmission of the virus from nurseries or finishing units [79].

It is generally acknowledged that most infections are subclinical in chronically infected herds. For instance, Bilodeau et al. [80] detected viral circulation in a farrow-to-finish farm using sentinel pigs several months after the cessation of the outbreak. The subclinical infection was also detected in a neighbouring barn that had never experienced clinical PRRS, and was situated 50 m from the main farm. Likewise, Stevenson et al. [76] monitored type 2-infected 6–8 week-old piglets of two farrow-to-finish farms that experienced a reproductive outbreak 2.5 years before. They found that most of the necropsied pigs were positive in lungs and spleen by viral isolation, confirming that PRRSV was circulating in nursery pigs despite both farms being clinically “healthy” for several years since the original outbreaks.

## Quantification of PRRSV transmission

The knowledge of the dynamics of PRRSV circulation within a herd and the quantification of the virus transmission in a pig population are key points for the development of prevention and control strategies of the infection, as well as for estimating the impact of such interventions. Unfortunately, there are currently only few studies available on this topic. The reproduction rate (R) is usually used for quantification purposes. R is defined as the mean number of cases infected by one infectious case [81], and is equal to the duration of the infectious period multiplied by the transmission parameter β (probability of contact between infectious and susceptible by probability of transmission in case of contact per unit of time). The higher the R-value, the greater and faster is the spread in the population. Moreover, when R < 1, the infection tends to fade-out with time in the assumption of a large and homogeneous population.

Nodelijk et al. [78] quantified the transmission within a herd using the serological data obtained from a longitudinal study of a type 1-infected closed breeding farm (115 sows) that experienced a major outbreak 6 years before. The results indicated that during the first wave of the epidemic, seroconversion was observed in 80% of the sows and 49% of rearing pigs, respectively. Four years after the epidemic, and until the end of the study none of the pigs seroconverted, and all sera were negative for PRRSV, indicating the total fade-out of the virus. In the same study, the reproduction rate was estimated to be 3.0 (CI_95%_ 1.5–6.0) for sows devoid of previous immunity, assuming that the infectious periods of pigs lasted 56 days, and that there was no life-long immunity after infection. Charpin et al. [35] estimated an R of 2.6 (CI_95%_ 1.8–3.3) for naive piglets, using an experimental model of transmission of type 1 PRRSV by contact between inoculated and susceptible pigs introduced at different times post-infection. In that same work, the mean duration of contagiousness was estimated to be 14.8 days, with a peak of infectivity at 9 dpi, and a negligible probability of transmission after 42 dpi, although inoculated piglets were positive using RT-PCR in sera up to 77 dpi.

The reproduction rate of a genotype 1 PRRSV was also assessed in growing pigs by our research group [82]. We used a model of transmission by contact that mimicked natural conditions: groups of five susceptible pigs (six replicates in total) were co-mingled and each left in contact with an experimentally inoculated pig for a period of 21 days. The development of viraemia was monitored using quantitative real time-PCR. Assuming the mean duration of viraemia as the infectious period of the animals belonging to the same contact group (including the inoculated pig), we estimated an R value of 2.78 (CI_95%_ 2.1–3.4) for growing pigs. More recently, Rose et al. [83] also performed an experiment to quantify the transmission of genotype 1 PRRSV in piglets. In this case, two susceptible pigs were kept in contact with two experimentally inoculated piglets (six replicates in total) for 49 days and the serum monitored by RT-PCR. They found that the mean period of contagiousness, calculated on the basis of the duration of viraemia, was 22.6 days, and that R for piglets was 5.4 (CI_95%_ 2.9–9.0).

Overall, it can be concluded that the R for genotype 1 PRRSV could range from approximately 2 to 5 in naïve pigs. Compared to other common swine pathogens, such as classical swine fever virus (R = 15) [84], PRRSV does not seem to be transmitted that easily. There are few values for genotype 2 or for subtypes 2–4 of genotype 1. In general, type 2 is thought to comprise strains of higher virulence [85] than type 1, at least for subtype 1.

## Vaccination and PRRSV transmission

Vaccination of sows and piglets is one of the strategies commonly used for controlling PRRS, together with management and biosecurity measures. At present, several commercial attenuated (modified live vaccines, MLV) or inactivated (IV) vaccines, based on both genotype 1 and genotype 2 PRRSV strains are available. Protection afforded by these vaccines has to be evaluated at both individual and population levels. In the first case, the main objective of vaccination is to protect pigs from the infection and reduce clinical signs, whereas at population level, the aim of vaccination strategies for controlling PRRS is also to reduce the economic losses associated with the disease and to stop virus transmission. Vaccination strategies with MLV are currently predominant. Modified live vaccines are able to replicate in the host, and induce an immune response similar to that induced by mildly virulent PRRSV isolates.

As mentioned above, the virological and clinical protection afforded by MLV vaccination is considered partial against a heterologous PRRSV strains; however, in general, vaccinated pigs experience a viraemia of shorter duration compared to naïve counterparts. In any case, given the genetic diversity of PRRSV [86] most, if not all, challenge situations in the field can be considered as heterologous.

Stadejek et al. [87] evaluated the efficacy of MLV vaccination in a farrow-to-finish herd where a Polish wild type PRRSV strain was circulating for several years prior to the start of the vaccination program of PRRS control. Twelve piglets of that farm were vaccinated with a genotype 1 MLV at 14 days of age, and then were followed-up until 132 days of life. At 68–92 days post-vaccination, only two pigs had become infected with the field strain, despite the fact that the MLV and the wild type of the farm were only 82.6% similar (ORF5). Similarly, Martelli et al. [88] assessed the efficacy of vaccination of piglets against natural exposure to a PRRSV field strain belonging to the Italian cluster of genotype 1 PRRSV (8% of identity with the ORF5 of the MLV). In the post-exposure period, wild type virus was only detected in 59% of the sera of vaccinated pigs, and also clinical signs were significantly reduced in vaccinated animals compared to the unvaccinated ones.

As previously commented, at a population level, the efficacy of current vaccines cannot be only evaluated in virological terms. In epidemiological terms, the goal of vaccination is also to stop or decrease viral transmission within a farm. The ceasing of virus transmission in a herd could be achieved by vaccination, if the MLV has the ability to reduce the susceptibility of pigs against the infection and, at the same time, is capable of reducing the contagiousness of the individuals that eventually became infected. In this sense, the potential efficacy of MLV vaccines in the field can be estimated indirectly, by assessing the biological parameters related to transmission (i.e. duration of viraemia and shedding of the virus, number of chronically infected pigs etc.), and directly, by determining the reproduction rate of PRRSV.

Cano et al. [89] demonstrated that repeated immunisations with MLV vaccine in pigs previously infected with a homologous isolate significantly reduced the number of persistently infected pigs at 127 dpi, and also reduced the viral shedding after 97 dpi, although this strategy was not capable of completely eliminating the circulation of the wild type virus. In a similar study, Linhares et al. [90] showed that vaccination of pigs after challenge with type 2 PRRSV significantly reduced viral shedding in oral fluids and the presence of virus in the air, although the magnitude and duration of viraemia in vaccinated pigs was similar to the unvaccinated ones. Cano et al. [91] also observed a reduction in PRRSV shedding, but not in the proportion of chronically infected pigs, for type 2 virus after heterologous vaccination.

Although the abovementioned works demonstrated the efficacy of mass vaccination in reducing the biological parameters related to the virus transmission, few studies have dealt with the assessment of R in vaccinated and naïve pigs. Nodelijk et al. [92] were the first to evaluate the effect of vaccination on PRRSV transmission. However, they used a genotype 2 MLV and a genotype 1 challenge virus (LV), thus being a worst-case scenario. The authors performed three different trials. In experiment A, 5 vaccinated (V) pigs and 5 non-vaccinated ones (NV) were challenged with LV and then co-mingled with another 5 V and 5 NV, respectively. In experiment B, 1 V pig was inoculated with LV and placed in contact with another 9 V pigs. The same protocol was used for NV pigs. Finally, in trial C, transmission of PRRSV in 10 pairs of vaccinated pigs was compared with 10 pairs of non-vaccinated pigs by means of multiple one-to-one experiments. Most vaccinated pigs (>60%) became infected in experiments A and B, and all of them became viraemic in experiment C. Thus, the R value in vaccinated pigs, as estimated from the pooled data of trials A and B, was 1.5 (CI_95%_ 0.7–44.8), whilst it could not be determined (infinite) for NV pigs. The study failed, therefore, in demonstrating a significant reduction of PRRSV transmission after vaccination. However, considering that the vaccine and challenge strains were of different genotypes and that pigs were inoculated intranasally, the R value was probably overestimated. This phenomenon is partially confirmed by the evidence that V inoculated pigs had a significantly longer duration of viraemia and higher viral loads compared to V pigs infected by contact.

Mondaca-Fernández et al. [93] performed another study on the efficacy of vaccination in reducing type 2 PRRSV transmission. In that case, animals were vaccinated with a genotype 2 MLV, and also challenged with a genotype 2 PRRSV, the MN-30100 isolate. The authors failed in their objective, since the challenge strain was found to be little contagious, as indicated by the lack of infection in the exposed NV pigs. Thus, the R values were 0.59 (CI_95%_ 0.13–3.21) and 0.26 (CI_95%_ 0.01–2.26) for the vaccinated and non-vaccinated groups of pigs, respectively. The difference was not significant, but considering the low transmissibility of the challenge strain, it can be argued that PRRSV transmission could not be properly assessed.

Our experimental study for assessing the PRRSV transmission by contact [82] was the first work in which a significant reduction of the R value in vaccinated pigs was demonstrated. In this case, 3 week-old piglets were inoculated with a genotype 1 MLV and divided in 8 groups of five pigs each, as described for unvaccinated piglets in a previous section of the present review. At 35 days post-vaccination, 14 NV pigs were experimentally inoculated with a genotype 1 PRRSV isolate that showed 93.4% of nucleotide similarity with the MLV. Two days later, one challenged pig was introduced into each contact group to expose them to the virus. After 21 days of contact, all NV became viraemic, while only 53% of V pigs were detected as such. Moreover, compared to NV pigs, V animals withstood 2 weeks more of contact with the inoculated pig before becoming infected. The R value was 2.78 (CI_95%_ 2.13–3.43) in NV pigs, and 0.53 (CI_95%_ 0.19–0.76) in V animals.

More recently, Rose et al. [83] also showed a significant reduction in PRRSV transmission in vaccinated pigs. In that study, 3 week-old piglets were inoculated with genotype 1 MLV, and 12 of them were then challenged with a genotype 1 strain (92.7% of sequence homology with the MLV) at 31 days post-vaccination. The inoculated pigs were then put in contact with 12 vaccinated piglets for 49 days (6 replicates of 2:2 contact trials in total). The experiment was replicated simultaneously with non-vaccinated piglets. Among the contact pigs, the challenge strain was detected in the serum of only one V, whereas all contact NV were infected. Consequently, the R was significantly reduced from 5.42 (CI_95%_ 2.94–9.04) in NV pigs to 0.30 (CI_95%_ 0.05–0.96) in V animals.

The results of the abovementioned studies [83, 84] have to be carefully interpreted since the use of other vaccines or challenge isolates may have produced numerically different results. In spite of this, the similar results point towards a potential use of mass vaccination to stop transmission of genotype 1 PRRSV.

It is worth to compare the abovementioned data with the information available for other important and common pig viruses. For example, in Aujeszky’s disease virus, de Jong et al. [94] estimated R equal to 10 for naïve pigs with a reduction up to R = 0.5 in vaccinated pigs. Stegeman et al. [95] estimated R to be equal to 3.4 in single vaccinated pigs and R = 1.5 in double vaccinated pigs and Bouma et al. [96] estimated R for Aujeszky’s disease virus vaccinated pigs to be <1. For swine influenza virus, R was estimated to be around 10 [97, 98] with a reduction of R up to 1 in vaccinated pigs [97]. This comparison of R-values in vaccinated pigs indicates that, at least for the classical genotype 1 subtype 1 isolates of PRRSV, moderate improvements in the vaccine efficacy may result in epidemiologically efficient strategies of vaccination.

## PRRSV transmission between farms

The virus may reach a farm in several ways, but entering infected animals, particularly gilts and sows, is considered the most common route for virus introduction [99–102]. For example, Mortensen et al. [101] associated the spread of type 2 PRRSV in Denmark to the purchase of infected animals without performing quarantine measures. Similarly, in a molecular study in Canada, Thakur et al. [102] found that the predominant relationship between strains within a cluster were the introduction of infected pigs into farms. Also in Canada, Kwong et al. [103] and Rosendal et al. [104] identified the source of gilts as one of the main causes for explaining the spread of different strains in the country.

The use of contaminated semen is also an important route for the introduction of PRRSV into a farm. For instance, Bøtner et al. [105] demonstrated that the clinical outbreaks occurring in Danish PRRS-free breeding herds in July of 1996 were caused by a genotype 2 isolate, previously unrecognised in that country. The virus was found to be 99% similar to the live vaccine used in boars since December 1995. Semen imported from Germany was also identified as the origin of the introduction of PRRSV in five Swiss herds in November 2012 [106]. Introduction of PRRSV into the farm by semen or by infected gilts is relatively easy to avoid if PRRSV-free sources are used, and adequate testing of boars and quarantine measures are applied.

Several works indicated that trucks, trailers, and other vehicles used for transporting pigs, animal products, feed, offal, and contaminated equipment, are a potential risk for the spread of PRRS. For example, Dee et al. [107] demonstrated that pigs may become infected after been housed for 2 h in trailers artificially contaminated with ≥10^3^ TCID_50_/mL of the genotype 2 MN-30100 PRRSV isolate. In the same study, transmission of PRRSV was also observed in 3/4 trials where two PRRSV-naïve sentinel pigs were placed for 2 h in a trailer previously contaminated by experimentally inoculated pigs. Another two works simulated common farm worker behaviour to assess transmission of PRRSV by fomites (boots and containers), vehicle sanitation, transport, and the movement of personnel [108, 109]. The results showed that the infectious virus can be isolated from the ventral surface of transport vehicles, the truck wash floor, the floor mat of the trailers, drivers’ boots, and also from the surface of various types of containers. When the study was conducted during the cold season (<0 °C), infectious virus was recovered from at least one sampling point in 5/10 replicates, and viral RNA was detected at all sampling points in 7/10 replicates [108]. Conversely, in warm weather (>15 °C) the detection rate significantly decreased, suggesting that temperature is critical for virus survival in fomites and therefore, it was assumed that the role of those fomites becomes more important during winter [109].

Treatment of vehicles by washing at high temperature (80 °C) followed by phenol disinfection and overnight drying, was effective for a complete sanitation of trailers. Alternatively, the use of a thermo-assisted drying and decontamination (TADD) system or glutaraldehyde fumigation had an equivalent efficacy to overnight drying for the complete trailer decontamination [110, 111].

Proximity of infected herds has been considered a hazard, resulting in an increased risk of introduction of the virus by aerosol transmission [101, 112, 113]. Nevertheless, the airborne transmission of PRRSV and its implication on the area spread of the disease seems to be dependent on the strain and on environmental factors. Torremorell et al. [114] experimentally demonstrated the airborne transmission of type 2 strain VR-2332 between pigs located 1 m apart, while transmission did not occur when the MN-1b isolate was used instead. Similarly, airborne transmission occurred at distances of 1–2.5 m using the MN-30100 and the VR-2402 isolates, respectively [115, 116].

When the aerosol transmission of MN-30100 and VR-2402 isolates was evaluated under field conditions, no positive air samples (by RT-PCR) or infection in susceptible pigs were observed at the different distances between the building where infected pigs were housed and the trailer of susceptible pigs [116, 117]. However, using a source population of 300 grower-finisher pigs experimentally inoculated with the MN-184 isolate, infectious virus was detected from the exhausted air of the facility up to 4.7 km from the infected herd [118]. Evidence of long-distance airborne dispersion of PRRSV up to 9.1 km was also demonstrated from a herd experimentally infected with the MN-184 strain, but not for the MN-1-18-2 and MN-1-26-2 PRRSV isolates [119]. In the same study, the viral load in air samples decreased from 10^4^ TCID_50_/mL in the proximity of the infected barn to 10^1^ TCID_50_/mL at 9.1 km from the source population, indicating increasing deposition and/or inactivation of the virus with distance. Also, directional winds of low velocity, low temperatures, high relative humidity, and low sunlight levels are factors favourable to the airborne spread [120]. In the conditions of the North American Midwest, [121], it has been shown that air filtration can help to reduce about 80% of PRRSV introductions in farms of Southern Minnesota and Northern Iowa (USA). Collectively, the abovementioned data indicated that airborne transmission of PRRSV occurs, but is highly influenced by climatic conditions and the strain, and therefore its epidemiological importance may be different in different locations.

The only species known to be susceptible to PRRSV is the pig, either domestic or feral. Only one report indicated a potential role of mallard ducks (*Anas platyrhynchos*) [122], but subsequent studies did not confirm this [123].

Infection have been confirmed by RT-PCR in wild boars of Italy [124], Germany [125], and Slovakia [126], while serological evidence has been reported in Croatia [127], France [128], Germany [129], and also United States [130]. The detection of PRRSV viruses in wild boars similar to commercial life vaccines indicates that the virus has been probably transmitted from domestic pigs to wild boars [125, 126]. Thus, the role of feral swine in PRRSV area spread could be considered of limited relevance.

As regards the role of arthropods, Schurrer et al. [131] demonstrated that houseflies can mechanically harbour the virus for up to 48 h, although they did not support PRRSV replication. Actually, contaminated flies were shown to be able to transmit the infection to susceptible pigs [132, 133]. Similarly, Otake et al. [134] demonstrated the transmission of PRRSV from experimentally inoculated pigs to susceptible animals in 2/4 trials. However, in spite of the potential significance of these data, cautious interpretation is advisable, since those studies used artificial exposure models that did not mimic field conditions. Moreover, movements of houseflies between farms are limited by several factors, including the existence of ventilation systems and filters, and the environmental conditions, such as temperature, relative humidity, and wind direction and speed [133]. Taken together, the data indicate that houseflies and mosquitoes can play a role in the spread of the virus within a limited radius [135, 136] and, in practical terms, this route has to be considered of minor importance.

The risk of introduction of PRRS in countries free of the disease through importation of contaminated meat and pork products has been also evaluated. In fact, pigs may become infected after ingestion of meat samples negative by virus isolation, but positive by means of RT-PCR [136, 137]. Nevertheless, after conventional post-slaughter handling and freezing, or after traditional manufacturing of pork products, the amount of infectious PRRSV in these products is very low, or even negligible [138, 139]. Therefore, the likelihood of importing the disease through meat imports is limited, but has to be taken into account [140, 141].

## Conclusions

The epidemiology of PRRSV is far from being fully elucidated, but the accumulated knowledge is enough to identify, at least qualitatively, the main sources of PRRSV infection of a farm, as well as the main mechanisms of transmission within the farm. What is still unknown is what proportion of virus introductions occurs by each route in different epidemiological scenarios. At least for genotype 1, the quantification of transmission indicates that PRRSV is less transmissible than other viral pathogens of swine, and this may permit transmission to be controlled by means of vaccination, even with the currently available vaccines that only afford a partial protection. A combination of strict biosecurity and rationally designed vaccination programs may be really useful to control PRRS in a farm or on a regional basis.
